# Radiofrequency Ablation of Thyroid Nodules: Basic Principles and Clinical Application

**DOI:** 10.1155/2012/919650

**Published:** 2012-10-22

**Authors:** Ji Hoon Shin, Jung Hwan Baek, Eun Ju Ha, Jeong Hyun Lee

**Affiliations:** Department of Radiology and Research Institute of Radiology, Asan Medical Center, University of Ulsan College of Medicine, Seoul 138-736, Republic of Korea

## Abstract

Radiofrequency (RF) ablation has been gaining popularity as a minimally invasive treatment for benign thyroid nodules regardless of the extent of the solid component. RF ablation of benign nodules demonstrated volume reductions of 33–58% after one month and 51–85% after six months, while solving nodule-related clinical problems. RF ablation has recently shown positive short-term results for locoregional control as well as symptom improvement in patients with recurrent thyroid cancers. This paper reviews the basic physics, indications, patient preparation, devices, procedures, clinical results, and complications of RF ablation.

## 1. Introduction

Thyroid nodules constitute a frequently seen clinical problem, and the incidence of thyroid nodules has increased with the recently increased use of thyroid ultrasonography (US) [1]. Although most thyroid nodules are benign and do not require treatment, some benign nodules may require treatment for associated symptoms and/or because of cosmetic problems [2, 3]. As curative surgery has several drawbacks [4] and the efficacy of thyroid hormone-suppressive therapy has not yet been determined [5], nonsurgical, minimally invasive treatment modalities, such as ethanol ablation (EA), percutaneous laser ablation, and radiofrequency (RF) ablation, have been used to treat thyroid nodules. EA is known to be very effective for treating cystic nodules, although it is less effective for solid nodules [2, 6]. Laser ablation has been investigated primarily for the treatment of solid nodules [7]. Recently high-intensity focused ultrasound and microwave have been introduced as thermal ablation methods for the treatment of thyroid nodules [8–10].

Since RF ablation of thyroid nodules was introduced in 2006, it has been reported to be both safe and effective for treating benign thyroid nodules and recurrent thyroid cancer. This paper provides information regarding the basic principles, indications, devices, and techniques that have been especially designed to optimize thyroid RF ablation, as well as the clinical results and complications. 

## 2. Basic Principles of RF Ablation

 In brief, RF ablation uses the heat generated from high-frequency alternating electric current oscillating between 200 and 1200 kHz [11]. The RF waves passing through the electrode agitate tissue ions around the electrode, and they increase the temperature (by frictional heat) within the tumor tissue, thus resulting in the destruction of tumor located very close, that is, within a few millimeters, to the electrode. In addition to the frictional heat, conduction heat from the ablated area can result in relatively slower damage of the tumor or to tissue remote from the electrode tip. This process of thermal injury secondary to friction and conduction heat is the basic mechanism of RF ablation [11, 12]. 

 At temperatures between 60 and 100°C, nearly immediate tissue coagulation is induced with irreversible damage caused to tumor tissue, while temperatures greater than 100–110°C result in tissue vaporization and carbonization which serve as an insulator to prevent heat spread and thus reduce the efficacy of RF ablation [11, 13]. The efficacy of RF ablation can also be reduced due to (1) the heterogeneous nature of the target tissue in the presence of fibrosis or calcification by altering electrical and heat conduction or (2) adjacent blood flow by perfusion-mediated tissue cooling [2]. 

## 3. Treatment Indications and Patient Preparation

 RF ablation can be used to treat both benign thyroid nodules and inoperable, recurrent thyroid cancers located in the surgical bed as well as lymph nodes [14–28]. Indications for RF ablation of benign thyroid nodules are nodule-related clinical problems such as symptoms, for example, neck pain, dysphasia, foreign body sensation, discomfort, and cough, cosmetic problems, or thyrotoxicosis in cases of autonomously functioning thyroid nodules (AFTNs) [29]. The Korean Society of Thyroid Radiology does not recommend thyroid RF ablation for follicular neoplasms or primary thyroid cancers because there is no evidence of a treatment benefit by RF ablation in follicular neoplasms or primary thyroid cancers [17, 29, 30]. Caution should be taken with regard to the use of thyroid RF ablation in pregnant women, patients with serious heart problems, and those with contralateral vocal cord palsy [29].

 According to the 2012 consensus statement and recommendations of the Korean Society of Thyroid Radiology [29], at least two separate US-guided fine needle aspirations and/or core needle biopsies are necessary to confirm the benign nature of a nodule [31, 32], and caution should be taken when performing RF ablation of nodules with malignant US features even when there are benign results seen on fine needle aspiration or core needle biopsy [33–36]. US examination is important for characterizing a nodule and to evaluate the surrounding anatomical structures [1]. The size, shape, margin, proportion of solid/cystic components, echogenicity, calcification, internal vascularity, and extracapsular invasion of each nodule should be evaluated [1]. Three orthogonal nodule diameters, including the largest diameter, should be measured by US, and the nodule volume could be calculated using the equation: *V* = *πabc*/6, where *V* is the volume, *a* is the maximum diameter, and *b* and *c* are the other two perpendicular diameters. 

 Laboratory tests usually include a complete blood count, a blood coagulation battery, and measurements of thyrotrophin, thyroid hormones, thyroid autoantibodies, and calcitonin. If any serum concentrations are abnormal, RF ablation should be performed only after performing procedures to correct these abnormal test results [29]. ^99m^Tc pertechnetate scintigraphy can be used to differentiate cold nodules from AFTNs, especially in patients showing decreased serum thyrotrophin concentrations. 

 Prior to treatment of recurrent thyroid cancers, tumor recurrence should be confirmed by positive US-guided fine needle aspiration cytology and measurements of the washout thyroglobulin (Tg) concentration [29]. US examination is important in order to evaluate the size and characteristics of a recurrent tumor as well as the critical surrounding anatomic structures. A neck CT may be used when appropriate for the evaluation of a recurrent tumor prior to RF ablation [29]. 

## 4. RF Ablation Devices and Procedures 

### 4.1. Devices for Thyroid RF Ablation

 As thyroid nodules are relatively large and elliptical in shape and have almost no safety margins, in contrast to liver tumors, electrodes for thyroid nodules are necessary and were developed in Korea. These modified, straight-type electrodes are short (7 cm), thus making them easy to control and thin (18 gauge), thus minimizing injury to the normal thyroid gland and can be used with active tips of various lengths, for example, 0.5, 0.7, 1.0, or 1.5 cm [22]. For example, small-sized (0.5 or 0.7 cm), active tips are effective for the RF ablation of small, recurrent thyroid cancers [23]. Ground pads (dispersive electrodes) applied to the skin are connected to the radiofrequency generator, and the generator is connected to the RF needle electrode. A peristaltic pump is used to perfuse chilled water (15–20°C) through the perfusion port of the electrode to prevent tissue charring and to improve the radius of RF energy deposition.

### 4.2. Procedures for Thyroid RF Ablation

 The patient is placed in the supine position with mild neck extension, after which a grounding pad is firmly attached to each thigh. 

**Figure 1:**
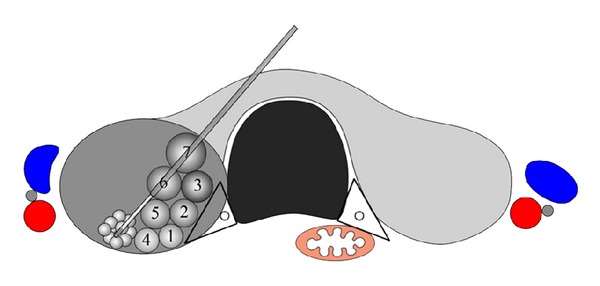
Schema of the transisthmic approach and the moving shot technique. The needle is inserted through the isthmus in order to visualize the entire length of the electrode and the target nodule. Ablation starts from the deepest portion of the nodule to the superficial area according to the order of the numbering of each small conceptual ablation unit, by moving the electrode tip. The ablation area is small near the peripheral danger triangle (black triangle), while it is large in the central, safe area. Recurrent laryngeal nerve (black circle) is within the danger triangle. The carotid artery (red color), internal jugular vein (blue color), and vagus nerve (gray color) are lateral to each thyroid lobe.

**Figure 2:**
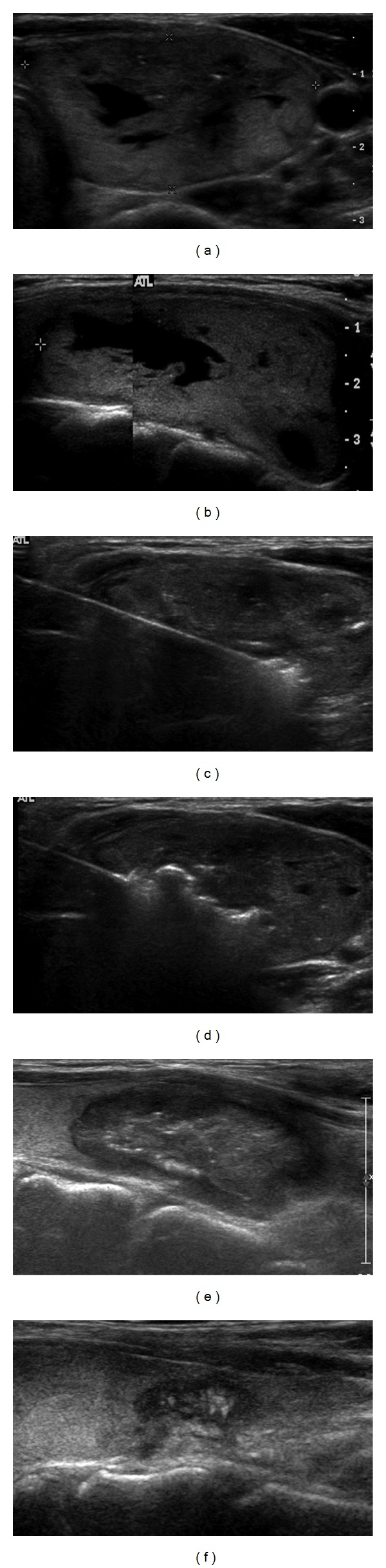
Sequential US images of benign thyroid nodules. (a), (b). Transverse (a) and longitudinal (b) US images show a predominantly solid nodule in the right lobe. The volume before treatment was 35 mL. (c). A transverse US image shows ablation of the periphery of a deep and remote portion of the nodule. The needle was inserted through the isthmus. (d). The transverse US image shows relocation of the electrode tip in the more central, untreated area. The second and third sessions of the RF ablation were performed within a one-year interval (not shown). (e), (f). Transverse US images one (e) and three-and-half (f) years following the initial RF ablation show progressive volume reduction. The final volume was 0.2 mL.

 The “transisthmic approach method” and the “moving shot technique” have recently been introduced [17, 22]. With the transisthmic approach, the electrode is inserted from the isthmus to the lateral aspect of a targeted nodule (Figures 1 and 2). The entire length of the electrode can be visualized via a transverse US view and with minimal heat exposure to the danger triangle which includes the recurrent laryngeal nerve and/or the esophagus. Secure positioning of the electrode through sufficient thyroid parenchyma also prevents leakage of ablated hot fluid outside the thyroid gland and change in the electrode position during swallowing or talking. 

 The moving shot technique was proposed by Baek et al. [17, 22] (Figures 1 and 2), as opposed to the fixed electrode technique which has been used to treat liver tumors. The fixed electrode technique is dangerous to surrounding critical structures because thyroid nodules are elliptical in shape. With the moving shot technique, multiple small conceptual ablation units are ablated unit-by-unit by moving the electrode. The electrode tip is initially positioned in the deepest and most remote portion of the nodule, after which it is moved backward to the superficial and nearest portion of the nodule so as to prevent visual disturbance caused by echogenic bubbles. 

 The RF power is 30–120 W depending on the size of the active tip and the internal characteristics of the nodules. Ablation is started with 30–50 W of RF power and is then increased in 10 W increments, if a transient echogenic zone does not form at the electrode tip within 5–10 seconds, to a maximum of 80–120 W. The RF power is reduced or turned off for several seconds if a patient experiences severe pain, and the ablation is finished when all conceptual ablation units have become transient echogenic zones. A danger triangle could remain undertreated because of its close approximation to a recurrent laryngeal nerve or to the esophagus. 

## 5. Clinical Results

### 5.1. RF Ablation of Benign Thyroid Nodules

 The efficacy of RF ablation for reducing nodule volume and relieving nodule-related clinical problems was confirmed by the prospective comparison study by Baek et al. and using a control group [15]. In five, representative studies, reduction of the nodule volume after RF ablation ranged from 33–58% at one month and from 51–85% at six months postablation [15–17, 20, 22] (Table 1). The greatest volume reduction is usually observed within the first month after RF ablation, and further volume reduction is gradually observed thereafter [15–17, 22] (Figure 2). Recently, the mean volume reduction rate based on 111 patients with 126 benign thyroid nodules has been reported to be 93.4% four years following RF ablation [37]. 

 For symptomatic cystic (<10% of solid component) nodules, EA should be the first-line treatment modality because of its similar safety and efficacy to those of RF ablation, the fewer number of treatment sessions, and its cost effectiveness; the volume reduction rate was quite similar; 93.1% in EA and 92.2% for RF ablation, as seen at the 6-month followup [21]. In another recent study based on 217 patients with cystic or predominantly cystic nodules, the volume reduction rate was 85.2% at the time of the one-year followup [6]. For treating predominantly cystic nodules (10% < solid component < 50%), RF ablation is also safe and effective for patients with incompletely resolved clinical problems due to the solid components remaining following EA, which indicates that RF ablation is effective in both solid and cystic thyroid nodules [3, 17, 19, 21, 38]. When the nodules were grouped into mainly cystic, mixed, and mainly solid nodules, the volume reduction was significantly higher for the mainly cystic nodules than for the other types, as seen at the one-month followup. However, at the six-month followup there was no significant difference in the volume in any of the three types [17]. 

 RF ablation is effective in patients with AFTN, as it reduces the nodule volume, improves nodule-related clinical problems, and corrects abnormal thyroid function [14, 16, 20, 22]. RF ablation of AFTNs requires greater effort in order to ablate the entire nodule, including the peripheral area, as untreated portions could interfere with improvements in abnormal thyroid functioning, induce regrowth of treated nodules, and usually require more treatment sessions. 

 Incomplete ablation of nodule margins due to the presence of adjacent critical structures allows marginal regrowth of treated nodules, especially for patients with AFTNs [22]. Although the moving shot technique can successfully prevent marginal regrowth in many patients, undertreated portions adjacent to the danger triangle as well as large-size nodules remain vulnerable to marginal regrowth following RF ablation [2, 16, 22]. A patient with a large thyroid nodule, for example, greater than 20 mL, may require additional RF ablation due to incomplete treatment and unresolved clinical problems [39]. 

 Thyroid functions are considered to be only minimally influenced by RF ablation, although several anecdotal cases of permanent hypothyroidism after RF ablation have been reported in patients with elevated levels of antithyroid peroxidase antibodies [22, 40]. The possible cause of hypothyroidism seems to be the progression of autoimmune thyroiditis associated with preexisting antibodies. In patients who have previously undergone thyroid lobectomy, RF ablation preserves thyroid functions and therefore seems definitively advantageous over surgery or radioiodine therapy for the treatment of symptomatic benign thyroid nodules [29].

 Compared with laser ablation, the long-term volume reduction rate of RF ablation was superior to that of laser ablation, that is, 90–92% in RF ablation versus 48% in laser ablation, as seen in the three-year follow-up data [37, 41]. RF ablation also seems to be safer than laser ablation [7].

### 5.2. RF Ablation of Recurrent Thyroid Cancers

 In patients with recurrent thyroid cancer and who have a high surgical risk or refuse repeated surgery, RF ablation can be effective for maintaining locoregional control of their cancer or in order to improve their cancer-related symptoms [23–26]. With RF ablation of recurrent thyroid cancers, the mean volume reduction has been reported to be 56–93% [23, 26], and with 42–58% of the nodules disappearing completely [23–25], 64% of the patients experiencing symptom improvement [26], and with the serum thyroglobulin concentration decreasing [23–26]. However, at present there is still no long-term follow-up data. 

## 6. Complications

 As various complications can occur during RF ablation, knowledge of the possible complications and suggested technical tips is important for safe ablation and proper management. In a recent multicenter study of 1459 patients, the complication rate after RF ablation was 3.3% and with a major complication rate of 1.4% [40]. 

 Pain is the most common patient complaint during RF ablation, although the pain decreases rapidly when the generator output is reduced or turned off. Pain is usually self-limited and few patients complain of intractable pain [17]. 

 Voice change is a serious complication of RF ablation and is likely due to injuries to the recurrent laryngeal nerve or hemorrhage. Thermal nerve injury may be prevented by using the moving shot technique and by undertreating the conceptual ablation units adjacent to the nerve, which is known as the danger triangle [15, 17, 19, 22]. Familiarity with the variations of the vagus nerve, for example, located adjacent to the thyroid gland, is also helpful in order to prevent nerve injury [3, 42]. 

 Hematomas can usually be controlled by compressing the neck for several minutes. Serious perithyroidal hemorrhage may be prevented by examining the perithyroidal vessels before inserting the electrode and with the use of small-bore electrodes [40]. Most hematomas completely disappear within one or two weeks. 

 Skin burn, mostly first degree, at the electrode puncture site is possible, especially when a thyroid nodule is large and the skin bulges. Skin color changes usually resolve within one week following the procedure and are without sequelae [18, 40]. 

 Nodule rupture presents with sudden neck bulging and pain during the follow-up period. It is secondary to the acute volume expansion of a nodule due to hemorrhage [40]. This complication can usually be managed conservatively with antibiotics and/or analgesics. 

## 7. Future Perspectives

 In the future, RF ablation could be a promising minimally invasive technique for the treatment of benign thyroid nodules and recurrent thyroid cancers. RF ablation could also be used for inoperable patients with primary thyroid cancers. For safe and effective RF ablation, the operators should be aware of the US anatomy of critical structures in the neck as well as various techniques and devices used for thyroid RF ablation.

Although esophageal or tracheal injury, heart problems caused by the RF current, and thermal injury to critical structures in the neck such as the cervical sympathetic nerve and spinal accessory nerve have not been reported, the operator should be aware of the significance of such possible complications, especially when a recurrent tumor is close to neck nerves.

 In order to prevent thermal injury to critical structures, injection of fluid between the target tumor and critical structures has been suggested when treating the liver [43]. This technique is also useful when treating recurrent thyroid cancers. For the treatment of recurrent thyroid cancers of the neck, the use of a unidirectional ablation electrode has been suggested as this partially insulated electrode could modify the direction of the ablation zone and finally achieve a half-moon-shaped ablation zone rather than the round ablation zone usually made by a conventional electrode [44].

 As the RF current passes through the heart during thyroid RF ablation, heart attacks and arrhythmias may be possible complications [45]. In order to prevent cardiac problems caused by the RF current, a bipolar electrode could be used, although it is not yet available for use in the thyroid gland. 

 As suggested for the treatment of liver tumors, a wet, internally cooled electrode may prevent carbonization and could thus enhance the ablation zone [46, 47]. In the treatment of thyroid tumors, a wet electrode may increase size of the ablation zone and reduce the ablation time.

 The combination of ethanol and the RF ablation technique has been used for treating liver and thyroid tumors [19, 38, 48, 49] as ethanol can increase the size of the ablation zone and also prevent carbonization during RF ablation. Therefore, a combination of ethanol and RF ablation can be considered as the one of the successful treatment options for thyroid nodules.

## 8. Conclusion

 RF ablation is a safe and effective alternative to surgery for treating benign, nonfunctioning, or autonomously functioning nodules as well as for recurrent thyroid cancers. Its efficacy can be maximized by complete ablation of the entire tumor margin, which is essential in order to prevent marginal regrowth and to effectively reduce the size of thyroid nodules. And in order to minimize the possibility of complications, it is important to consider the broad spectrum of possible complications as well as the available preventative techniques. 

## Figures and Tables

**Table 1:** Characteristics and treatment results of radiofrequency ablation for benign thyroid nodules.

	No. of Pts/nodules	Nodule		Volume change				Session (mean)	Electrode type
		Type	Solid component (%)	V init. (mL)	VR1 (%)	VR6 (%)	VR last (%)
Jeong et al., 2008 [17]	236/302	Cold	0–100	6.13	58	85	84	1–6 (1.4)	I.C.
Baek et al., 2010 [15]	15/15	Cold	>50	7.5	49	80	—	1	I.C.
Baek et al., 2009 [22]	9/9	AFTN	60–100	15.0	36	71	75	1–4 (2.2)	I.C.
Deandrea et al., 2008 [16]	31/33	Cold + AFTN	>30	27.7	33	51	—	1	M.E.
Spiezia et al., 2009 [20]	94/94	Cold + AFTN	>30	24.5	54	—	79	1–3 (1.4)	M.E.

AFTN: autonomously functioning thyroid nodule; V init.: initial volume before RF ablation; VR1, VR6, and VR last: volume reduction at one and six months and on the last followup, respectively; I.C.: internally cooled; M.E.: multitined expandable.
